# Predictors for Poor Outcomes at Six Months on Pain, Disability, Psychological and Health Status in Greek Patients with Chronic Low Back Pain After Receiving Physiotherapy: A Prospective Cohort Study

**DOI:** 10.3390/clinpract15030063

**Published:** 2025-03-16

**Authors:** Matthaios Petrelis, Georgios Krekoukias, Ioannis Michopoulos, Vasileios Nikolaou, Konstantinos Soultanis

**Affiliations:** 1 1st Department of Orthopaedics, School of Medicine, National & Kapodistrian University of Athens, 12462 Athens, Greece; konsoultan@gmail.com; 2 Laboratory of Advanced Physiotherapy, Physiotherapy Department, School of Health & Care Sciences, University of West Attica, 12243 Egaleo, Greece; gkrekoukias@uniwa.gr; 3 2nd Department of Psychiatry, School of Medicine, National & Kapodistrian University of Athens, 12462 Athens, Greece; imihopou@med.uoa.gr; 4 2nd Department of Orthopaedics, School of Medicine, National & Kapodistrian University of Athens, 14233 Athens, Greece; vassilios.nikolaou@gmail.com

**Keywords:** chronic low back pain, predictors, disability, quality of life, somatic symptom disorders, pain, anxiety

## Abstract

**Background:** Although previous studies have suggested a variety of sociodemographic and psychological factors as predictors of poor outcomes in patients with chronic low back pain (CLBP), longitudinal studies remain rare. **Objectives**: To examine the prognostic indicators for poor outcome at 6 months on pain, disability, quality of life, anxiety, depression and somatic symptom disorders (SSDs) in Greek backache patients and to evaluate the medium-term effects of a conservative physiotherapeutic approach (massage, ultrasound, transcutaneous electrical nerve stimulation, low-level laser and exercise program). **Methods**: A prospective cohort study of 145 volunteers receiving treatment for CLBP in a physiotherapy unit was conducted using random systematic sampling. The intervention was assessed by comparing pre-treatment, post-treatment and six-month measurements with Friedman’s test and the Bonferroni correction, using the pain numerical rating scale (PNRS), Roland–Morris disability questionnaire (RMDQ), EuroQol-5-dimension-5-level (EQ-5D-5L), Hospital Anxiety and Depression Scale (HADS) and Somatic Symptom Scale-8 (SSS-8). Multiple linear regression analysis was carried out to determine the impact of demographics and pre-treatment scores with scores at six months. **Results:** The mean age was 60.6 years (±14.7). Post-treatment, statistically significant improvements were observed across all outcome measures, including PNRS, RMDQ, EQ-5D-5L and SSS-8 (all *p* ≤ 0.001), with anxiety showing a notable reduction (*p* = 0.002). After examining the multiple regression analysis, pre-treatment SSS-8 emerged as a predictor of elevated levels of pain, disability, anxiety and depression at 6 months. **Conclusions**: The findings yielded not only somatic symptom burden, greater age and pain intensity as prognostic indicators for poor outcomes at six months, but also reported favorable medium-term effects for a conventional physiotherapy regimen in CLBP management, as well.

## 1. Introduction

In the 2021 Global Burden of Diseases (GBD) study, low back pain (LBP) was among the ten top causes of disease burden, having as a consequence 70.2 disability-adjusted life years (DALYs) for both sexes [1]. In Greece, it has been observed as a consistent reason for years lived with disability (YLDs), highlighting LBP as one of the five foremost causes of YLDs during 2000–2016 [2]. The etiology of LBP is largely unknown, and in the event that the pain lasts more than 3 months, it is characterized as chronic LBP (CLBP). Literature findings have shown that CLBP is associated with high values of pain sensation, significant limitation in activities of daily life (ADLs), impaired health-related quality of life (HRQoL), severe anxious, depressive and somatic symptoms (which in turn lead to increased socioeconomic costs due to healthcare consumption), absenteeism and loss of productivity [2,3,4,5,6,7]. For that reason, the burden of CLBP is a major public health problem, for which health policymakers should pay specific attention and for which researchers should intensify their concerted research efforts in the context of a biopsychosocial model [3,8].

According to the clinical guidelines and latest systematic reviews, non-pharmacological approaches are key features of CLBP management, involving a diversity of interventions, such as supervised exercises, physical therapy modalities and cognitive behavioral therapy to alleviate the unpleasant symptoms cited before [9,10,11]. In particular, these guidelines endorse massage and exercise as first-line or adjunctive treatment options for persistent LBP [12,13]. Furthermore, it is well documented that a combination of physical therapist-applied treatment, like an exercise program, therapeutic ultrasound (US), massage, transcutaneous electrical nerve stimulation (TENS) and low-level laser therapy, have favorable effects on pain intensity, quality of life, functional disability and psychological state in the short term [10,11,14,15,16,17,18,19]. However, the effectiveness of conventional physiotherapy interventions for CLBP has not been demonstrated beyond a reasonable doubt. Regarding a systematic review of 83 randomized controlled trials, there is conflicting evidence on the efficacy of the aforementioned approach for the treatment of CLBP, suggesting that further research should be conducted to better comprehend and compare its effectiveness [20,21]. Additionally, previous studies have mentioned a diversity of demographic and psychosocial factors that are considered prognostic indicators of poor outcomes in backache patients receiving physiotherapy [22,23]. Particularly, Karstens et al. [22] noted in their prospective cohort study that age, impairment and pain prior to intervention, as well as mental disorders and physical activity, are outstanding predictors of disability at 6 months after the end of a physiotherapy program. Similarly, in a recent cohort study of 115 patients with non-specific LBP looking for primary care, Cruz et al. [23] implied that patients with CLBP, who had greater maladaptive psychosocial scores and who were unemployed had an increased probability of worse pain and disability levels. Nevertheless, there is a paucity of longitudinal studies that have adequately investigated the medium-term effects of the conservative treatment of CLBP, and that have identified the predictive role of the factors mentioned before with the 6-month scores of patients in the Greek population. Namely, Zografakis-Sfakianakis et al. [24], in their prospective study of 80 LBP patients receiving a combination of different physical modalities and exercise, partially examined the substantial enhancement in HRQoL after only one month of follow-up.

The objective of the present study was to widely examine the prognostic indicators for poor outcomes at 6 months on pain, disability, quality of life, anxiety, depression and somatic symptom disorders (SSD) in Greek CLBP patients, and to evaluate the medium-term effects of an equivalent intervention for the first time. We hypothesized that (1) sociodemographic and pre-treatment scores of pain, disability, anxiety, depression, SSD and HRQoL would be associated with the scores at 6 months after receiving physiotherapy, and that (2) a physiotherapeutic program would demonstrate satisfactory results in reducing pain severity, reducing functional disability and increasing psychological well-being and health status.

## 2. Materials and Methods

### 2.1. Study Design and Participants

Bearing in mind that placebo control was not morally recommendable and the obtainable patient pool was restricted, we conducted a prospective cohort study between April 2021 and November 2023 in consecutive patients with CLBP, visiting the outpatient physical therapy department of TYPET (Greek acronym for the Mutual Fund of National Bank of Greece Personnel) in Athens, Greece. We had access to their medical records and used a random systematic sampling method to enroll participants; every second patient was asked to complete a form. For the intervention part of the study, the power analysis methodology represented a design, with three levels of the within-subject factor of time. For this design, 130 participants achieved a power of 0.95 for the within-subject main effect at an effect size of 0.14 or more. The calculated sample was increased to 145 in case any participants were lost to follow-up. Of a total of 290 eligible patients with CLBP, 145 participants were enrolled in the survey by the researchers. The inclusion criteria encompassed suffering from CLBP (pain, discomfort and stiffness at T12 or lower with or without lumbar radiculopathy) and sufficient Greek language skills. Exclusion criteria were having had physiotherapy in the last six months, pregnancy, spinal cancer, surgery, a fracture, psoriatic or rheumatoid arthritis, ankylosing spondylitis, spondylolisthesis, cauda equina syndrome, or scoliosis more than 20° (red flags). All volunteers were informed by the researchers and signed a written consent questionnaire before their participation in the survey, and the survey adopted the principles of anonymity and confidentiality.

The research was authorized by the School of Medicine of the National and Kapodistrian University of Athens (protocol number: 1920031321/27-07-2020) and the medical ethics board of primary healthcare services of TYPET (protocol number: 005294/19-10-2020). The study adhered to the principles of the Declaration of Helsinki, following the Strengthening the Reporting of Observational Studies in Epidemiology (STROBE) statement for reporting cohort studies [25,26]. This was a prospective study, and its protocol was registered in an international database (www.anzctr.org.au/ number 12623000921684, accessed on 28 October 2024).

The conventional physiotherapy treatment was performed by two experienced physiotherapists and applied to the paravertebral lumbosacral region (one-on-one) for a total of ten sessions (five times a week). The parts of the treatment package can be found elsewhere [18,19,27,28,29,30,31]; treatment lasted about 60 min and consisted of a combination of continuous ultrasound for 5 min (frequency: 1 MHz, intensity: 1.5 W/cm^2^), 15 min of massage (techniques of rolling, wringing, deep stroking, pulling, friction), TENS for 20 min with 4 electrodes, 20 min with a set of 10 repetitions of standardized exercises for strengthening and flexibility (back extension and lower abdominal exercises, cat and camel, pelvic tilt, knee to chest, supine plank, abdominal hollowing, bird and dog, oblique crunch) and continuous low-level laser therapy (830 nm; 120 m; 0–50,000 Hz). We chose to use usual care or common practice because it was assumed that the variability in treatments applied washed out specific treatment modifier effects [32]. In order to handle position intolerance, subjects could choose to lie down on a bed or sit in a chair during the treatment. All individuals were oriented to adopt an active role and continue the same exercise program (one set per day) after the end of the treatment and throughout the follow-up period, emphasizing the importance of compliance.

### 2.2. Measures

All participants were assessed before the first session (baseline), at the end of the tenth session (post-treatment) and then 6 months later using a paper-and-pencil questionnaire. This form incorporated items on sociodemographic characteristics (gender, age, height, body weight, work and marital status, physical activity) and widely used patient-reported outcome measures for pain, disability, HRQoL, anxiety, depression and SSD. The measures are described below:

Body mass index (BMI) is an indicator of total body fat (body weight in kilograms divided by the square of the height in meters), defining four classifications: underweight (≤18.5 kg/m^2^), normal weight (18.5–24.9 kg/m^2^), overweight (25.5–29.9 kg/m^2^) and obese (≥30 k/m^2^) [28].

The pain numerical rating scale (PNRS) is a measure of pain intensity (current, best and worst level over the last 24 h), ranging from 0 (no pain) to 10 (worst pain you can imagine) [33].

The Roland–Morris Disability Questionnaire (RMDQ) contains 24 items, evaluating the degree of activity limitations due to LBP, and varying from 0 to 24, with higher values indicating greater levels of disability [34].

The commonly used 5-level version of the EuroQol-5D questionnaire assesses HRQoL according to responses in a descriptive system of five dimensions (mobility, self-care, usual activities, pain/discomfort, anxiety/depression), classifying 3125 unique elements of health status, ranging from 11,111 (perfect health level) to 55,555 (worst health level) [35,36].

The Hospital Anxiety and Depression Scale (HADS) is a valid and reliable questionnaire of fourteen items (seven items for each subscale) on a 4-point Likert scale that estimates the severity of anxiety and depression. The sum of points is between 0 and 21, with higher scores indicating a higher burden of anxiety and depression [37].

The new Somatic Symptom Scale-8 (SSS-8) is a diagnostic tool for monitoring the degree of SSDs during the past week using a five-point Likert scale. Total scores vary from 0 to 32, with higher values denoting greater somatic symptom severity [38].

The reliability and validity of the aforementioned measures have been previously confirmed within the Greek population, and they have been recommended for use in patients with CLBP [39,40,41,42].

### 2.3. Statistical Analysis

Quantitative variables were expressed as mean values with standard deviations and as medians with interquartile ranges, while categorical variables were expressed as absolute and relative frequencies. Friedman’s test was used for score comparisons between pre-treatment, post-treatment and 6-month measurements. The Bonferroni correction was used in order to control for type I errors. The effect size estimate Kendall’s W value for Friedman’s test was computed via the formula W = χ2/N(K − 1), where χ2 is the Friedman’s test statistic value, N is the sample size and K is the number of measurements [43]. Kendall’s W uses Cohen’s interpretation guidelines of 0.1–0.29 (small effect), 0.3–0.49 (moderate effect) and ≥0.5 (large effect) [44]. Multiple linear regression analysis was used, with the scores at 6 months used as the dependent variable. The regression equation included terms for demographic information and pre-treatment scores. Adjusted regression coefficients (β) with standard errors (SEs) were computed from the results of the linear regression analyses. Logarithmic transformations of the dependent variables were used due to the lack of normal distribution. All reported *p* values are two-tailed. Statistical significance was set at *p* < 0.05, and analyses were conducted using SPSS statistical software (version 26.0).

## 3. Results

**Figure 1:**
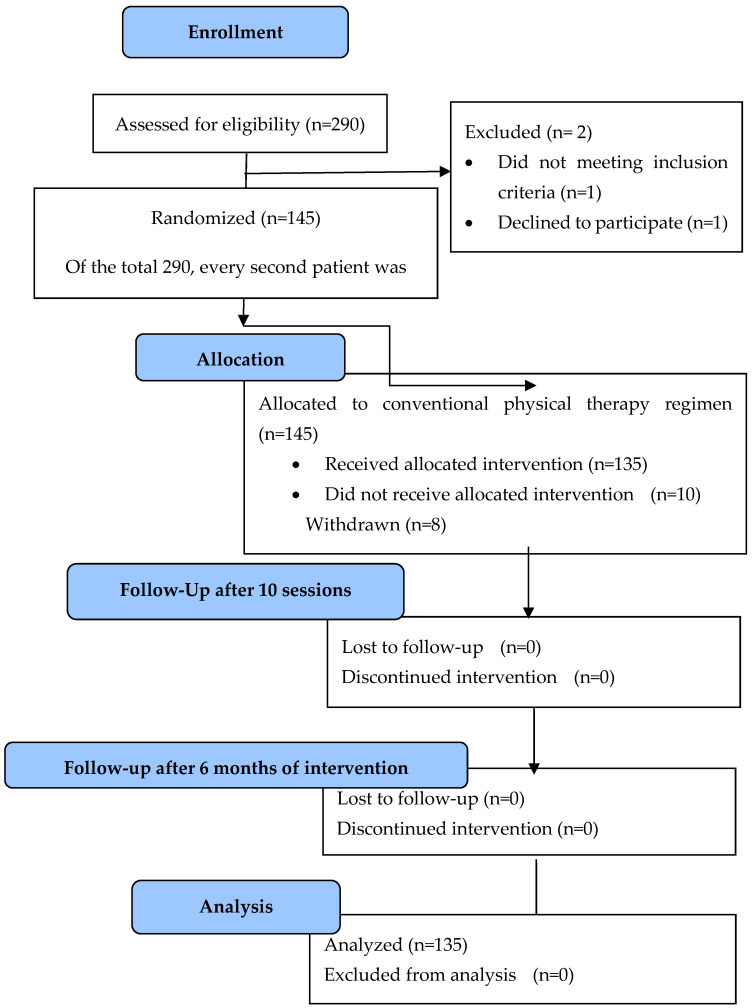
Flow diagram of the study.

Initially, out of a total of 290 eligible CLBP patients, 145 participants were randomized and enrolled in the study (Figure 1). Finally, the sample consisted of 135 participants (93.1% response rate, 60% women), with a mean age of 60.6 years (SD = 14.7 years). Mean BMI was 27.2 kg/m^2^ (SD = 5.3 kg/m^2^), and 41.5% were overweight. In the sample, 54.8% were married, 41.5% were university alumni and 61.5% were retired (as depicted in Table 1).

After multiple linear regression analysis, it was found that a greater age (β = −0.004; *p* = 0.001) and greater pre-treatment pain score (β = −0.027 *p* < 0.001) were significantly associated with a lower EQ-5D score at 6 months (Table 2). Also, only the pre-treatment SSS-8 score was significantly associated with the HADS scales at 6 months (Table 3). More specifically, a greater pre-treatment SSS-8 score was associated with greater depression (β = 0.018; *p* < 0.001) and with greater anxiety (β = 0.017; *p* = 0.008) scores at 6 months. Furthermore, a greater age (β = 0.009; *p* = 0.006), greater pre-treatment SSS-8 (β = 0.016; *p* = 0.025) and a greater pain score (β = 0.036; *p* = 0.024) were significantly associated with a greater RMDQ score at 6 months (Table 4). A pain score at 6 months was significantly and positively associated with only the pre-treatment SSS-8 score (β = 0.019; *p* < 0.001). The SSS-8 score at 6 months was only found to be significantly associated with participants’ BMI. More analytically, overweight participants had significantly lower SSS-8 scores at 6 months, indicating a lower somatic symptom burden than obese participants (β = −0.139; *p* = 0. 023).

**Figure 2:**
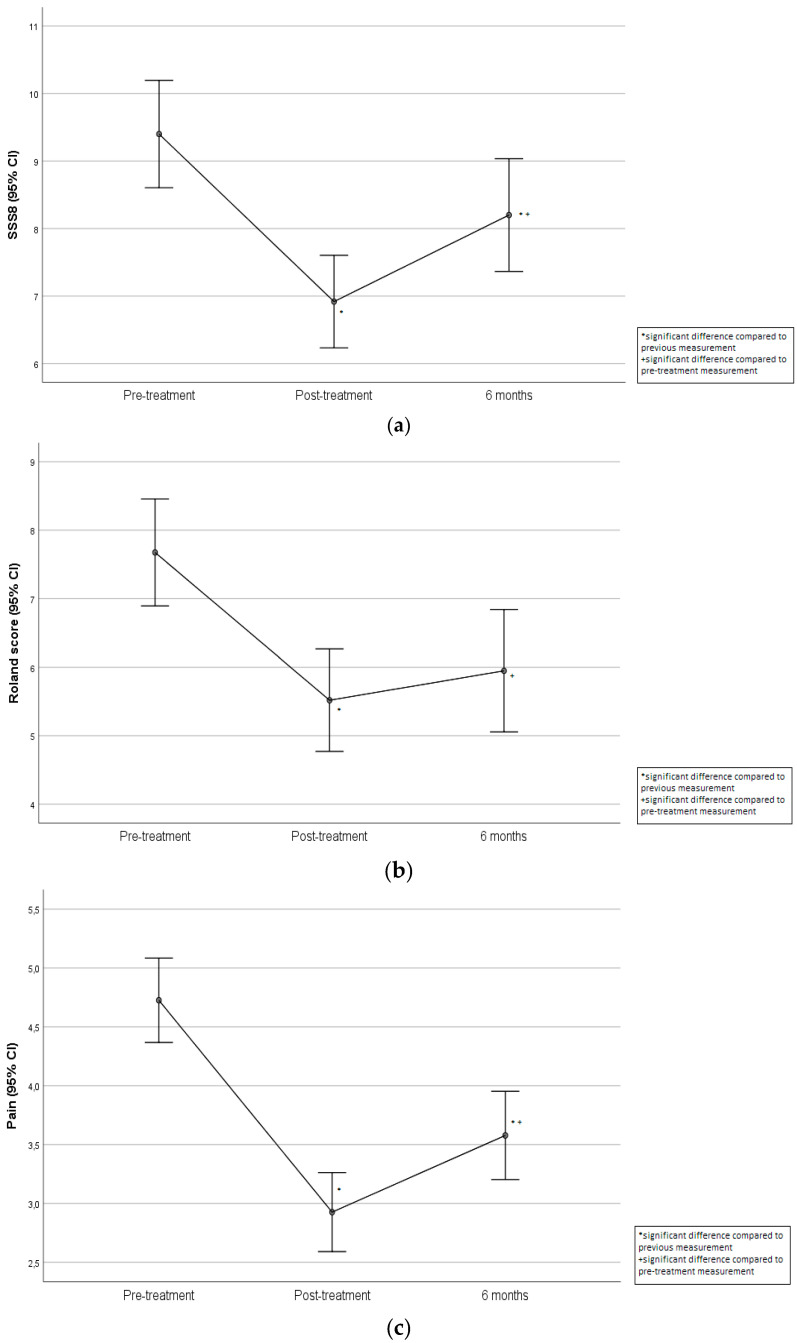

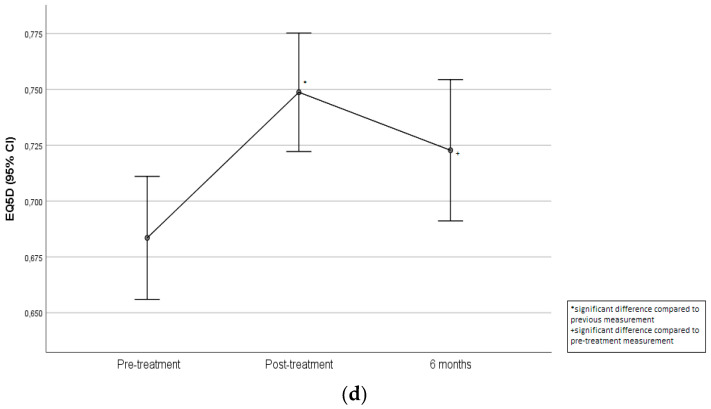
Changes in SSS-8 (**a**), Rolland–Morris (**b**), pain (**c**) and EQ-5D-5L (**d**) scores throughout the follow-up period.

The changes in the SSS-8, HADS, RMDQ, EQ-5D-5L and pain scales throughout the follow-up period are presented in Table 5. The SSS-8 score changed significantly over the follow-up period. More specifically, after the Bonferroni correction, it was found that the pre-treatment SSS-8 scores were significantly greater than post-treatment scores (*p* < 0.001) and 6-months-after scores (*p* < 0.001). Also, post-treatment SSS-8 scores were significantly lower compared to 6-months-after scores (*p* = 0.001), (Figure 2a).

The depression scores tended to change, while the anxiety scores significantly changed, over the follow-up period. More specifically, after the Bonferroni correction, it was found that pre-treatment anxiety was significantly greater than post-treatment (*p* = 0.002). At 6 months, no significant differences were found compared to the pre- and post-treatment scores (*p* > 0.05).

The RMDQ, EQ-5D-5L and pain scores changed significantly over the follow-up period. More specifically, after the Bonferroni correction, it was found that the RMDQ and pain scores were significantly lower post-treatment (*p* < 0.001 for both scores) and at 6 months (*p* < 0.001 for both scores), compared to pre-treatment (Figure 2b,c). However, at 6 months, the pain levels were significantly increased compared to after treatment (*p* = 0.001), yet were still lower than the initial scores. On the contrary, EQ-5D-5L value was significantly greater post-treatment (*p* < 0.001), as well as at 6 months (*p* < 0.001), compared to pre-treatment (Figure 2d). A medium effect size was only found in the pain score, while in the rest of the scales the effect sizes were small.

## 4. Discussion

To the best of our knowledge, this was the first longitudinal study in Greek CLBP patients examining not only prognostic indicators for poor outcomes six months after receiving physiotherapy but also examining the medium-term effects on pain, disability, HRQoL, anxiety, depression and SSD, as well. Overall, the findings yielded that somatic symptom burden was significantly associated with higher anxiety, depression, pain and disability levels at six months. Besides SSD, greater age and pain scores were significantly and positively correlated with functional disability and, in addition, significantly related to worse HRQoL scores at the six-month follow-up. Furthermore, it was demonstrated that pain intensity, functional disability, somatic symptom severity and HRQoL were significantly and favorably changed and persisted until the 6-month follow-up after treatment when compared with baseline scores.

It is well established that baseline elevated scores of pain intensity, disability and psychological factors (anxiety, depression, SSD), as well as sociodemographic characteristics (gender, age, BMI, work and marital status, physical activity) are determinants of poor HRQoL in CLBP patients [6,45,46,47,48]. Notably, Tom et al. [6] showed that gender (being female), an older age, unemployment, low physical activity, greater pain intensity, disability and anxiety predict impairment in HRQoL. Ailliet et al. [48], in their longitudinal study among 591 CLBP patients, found that only higher scores of baseline somatization demonstrated positive and significant relations with elevated scores of pain and disability throughout the follow-up period. In addition, a prospective cohort study of 6316 participants with CLBP showed that pain and disability had inverse correlations with quality of life at the six-month follow-up [46], while Keely et al. [45], using the baseline scores of HADS, reported that CLBP sufferers with anxiety and depression had a significantly lower HRQoL six months later. Apart from methodological differences and heterogeneous reference measures, the current study adds to this body of knowledge, showing less significant, but still distinct, correlations between age and pain intensity versus HRQoL. Furthermore, although it is generally recognized that somatic symptom burden is cross-sectionally associated with reduced HRQoL, we were not able to replicate this finding in the current longitudinal study [7,47].

According to our findings, the somatic symptom burden was the only psychological variable that seemed to predict pain severity and disability at six months, while greater age and pain intensity were identified as prognostic indicators of severe disability. These results are somewhat consistent with previous prospective cohort studies and a systematic review in individuals with CLBP who followed a conventional physiotherapeutic program using various PRO measures (a subscale of the Keele Start Back Screening Tool for anxiety, a subscale of the Symptom Check-List-90 for SSD, the Musculoskeletal Function Assessment Questionnaire for impairment in daily life, the Chronic Graded Pain Questionnaire and the German Pain Questionnaire for disability and pain) [22,23,49,50]. Namely, Karstens et al. [22] noted physical activity, mental disorders, pain and impairment prior to the intervention as considerable and positive contributors of disability across a sample of 792 patients with CLBP treated by physical therapists. An equivalent tendency was also revealed in a prospective cohort study Cruz et al. [23] of 115 individuals with LBP and a systematic review by Alhowimel et al. [50], which reported that greater maladaptive psychosocial variables (including anxiety and depression) and unemployment led to poor pain and disability outcomes six months after treatment. Lastly, like our findings, a longitudinal study of 484 chronic low backache patients highlighted that age and severe somatic symptoms pre-treatment were substantially correlated with increased disability at the one-year follow-up [49].

Furthermore, a novel finding of our study was the significant association of baseline somatic symptom burden with severe depressive and anxious symptoms at six months. Our results strengthen previous cross-sectional findings in individuals with CLBP and in the general population that somatization is positively correlated with anxiety and depression [49,51]. On the other hand, obesity seemed to be the only predictor of higher SSD scores at the follow-up, which is partially in line with other studies in general populations [52,53]. In particular, a review by Lykouras [52] and a Danish cross-sectional study of 5286 participants [53] noticed that obese individuals displayed significantly greater somatic symptom severity than the other BMI groups.

Our partially discrepant results may mirror the greater mean age and lower baseline anxiety, depression, SSD, pain and disability levels of the study sample and their prognostic role in poor outcomes in chronic low backache patients [22,23]. A potential explanation for our sample’s lower scores might be the “paradoxical” pattern of age effects, which states that older CLBP sufferers scored better on psychological well-being (anxiety and depression) and HRQoL [54]. Moreover, the lack of concurrent evaluations for anxious, depressive and somatic symptoms in these studies, despite their reporting overlap and comorbidities [47,48,55], as well as the existence of various differences in statistical methods—like participant characteristics, study sizes and sampling methods, durations of interventions and dissimilar PRO measures—might clarify these findings, since the selection of predictor variables are crucial for the outcomes of multiple regression analyses [49,56].

Additionally, it is generally recognized that the implementation of a physical agent modalities program with exercise in individuals with CLBP has beneficial effects on pain intensity, functional disability, HRQoL, and anxious, depressive and somatic symptom burden in the medium term, which we were able to replicate in our study [19,24,57,58,59,60]. Parallel to the literature, the current prospective study was the first in Greek CLBP patients in which a favorable and significant change in pain severity, functional disability, somatic symptom burden and HRQoL was observed at the 6-month follow-up after treatment, with small to medium (only in pain score) effect sizes for all scales. Similarly, a prospective Greek study of 80 backache inpatients previously demonstrated a clinically significant improvement in quality of life one month after the end of a conservative treatment [24]. Likewise, by applying physiotherapy methods focused on TENS, ultrasound, hot pack, exercise and medication in 50 subjects with CLBP (a total of 10 sessions, five times per week), Sahin et al. [19] reported a significant decrease in pain and disability scores (using the Visual Analog Scale [VAS] and Oswestry Disability Index [ODI]) at all intervals (pre- to post-treatment and at the three-month and one-year follow-ups). Thus, our less pronounced findings (small to medium effect sizes) are consistent with Konstadinovic and collaborators [58]—who examined the effectiveness of two different individual kinesiotherapy programs with physical therapy modalities (TENS and laser therapy) with ten sessions over two weeks in 80 CLBP patients—who found that pain and disability levels were alleviated during post-treatment, at the four- and eight-week follow-ups and with large effect sizes. Congruent with our results, longitudinal data from Brazil (a sample of sixty-six chronic low backache patients, divided into two groups: physiotherapy exercise and graded activity) pointed out that both interventions (a sum of 12 sessions, twice per week) had significantly favorable differences in pain intensity, functional disability and HRQoL in the short-term (post-treatment) and the medium-term (three- and six-month follow-ups) [57]. Moreover, an equivalent tendency was also revealed in a Spanish prospective cohort study of 120 participants with CLBP (control group), who received a routine physical therapy regimen, including TENS, microwave treatment and standardized exercises (a sum of 15 sessions, twice per week), inferring that such an intervention seemed to be a beneficial (but not significantly beneficial) option for improving functional disability and quality of life throughout the follow-up period (two, six and twelve months) [59]. Contrary to our results, Hampel and Tlach [60], in their two-year longitudinal study of 40 inpatients with CLBP following a conventional physiotherapy treatment (control group), detected substantial beneficial effects on anxious, depressive and somatic symptoms immediately after the intervention but not at the six-month follow-up assessment. These inconsistencies might be attributed to methodological differences in study sizes, interventions, duration of treatments and the PRO questionnaires for pain, depression, anxiety, SSD and HRQoL.

The present study had a few inherent limitations. First, the conclusions are limited to a single primary healthcare center in Athens (insufficient for a representative sample) and should not be generalized without caution to the general Greek population or to different clinical settings. Second, the over-representation of females, the absence of a control group and the absence of a check on what participants did with the exercise program after the end of the treatment may have influenced the results drawn from the study and limited their generalizability. Third, there was no blinding in our study because of the lack of a parallel control group, which may have introduced bias in interpreting the results. Last but not least, our sample consisted of a high proportion of pensioners, resulting in a mean age of 60.6 years. According to Wettstein et al. [54], older individuals with CLBP may report higher HRQoL and lower anxiety and depression scores than younger patients due to the “paradoxical” pattern of age effects, which may influence the impact of the prognostic indicators of poor outcomes and, by extension, the generalizability of our findings.

## 5. Conclusions

In summary, our findings strengthen the results of existing prospective studies in CLBP patients, yielding SSD as a significant prognostic indicator for greater levels of pain, anxiety, depression and disability at six months following treatment. Furthermore, greater age and pain levels were found to be considerable contributors to severe disability and poor HRQoL at the six-month follow-up. Additionally, the present study provides important evidence for the favorable medium-term effects (throughout the 6-month follow-up) of a conventional physiotherapy regimen for CLBP management, particularly pain intensity, functional disability, somatic symptom severity and HRQoL. Further large and longitudinal studies are required to confirm which psychosocial factors are predictors of poor outcomes and clarify the long-term effects of a conservative physiotherapeutic approach to CLBP.

## Figures and Tables

**Table 1:** Sample characteristics.

*n* = 135		Mean	SD
Age		60.6	14.7
BMI		27.2	5.3
		*n*	%
Gender			
	Man	54	40.0
	Woman	81	60.0
BMI			
	Normal	48	35.6
	Overweight	56	41.5
	Obese	31	23.0
Marital status			
	Unmarried	24	17.8
	Married	74	54.8
	Divorced	17	12.6
	Widowed	20	14.8
Educational level			
	Primary school	5	3.7
	Secondary school	8	5.9
	High school	36	26.7
	Two years of college	5	3.7
	University	56	41.5
	Postgraduate degree	25	18.5
Working status			
	Employee in the private sector	33	24.4
	Freelancer	6	4.4
	Employee in the public sector	0	0.0
	Pensioner	83	61.5
	Student	1	0.7
	Household labor	10	7.4
	Unemployed	2	1.5
	Unable to work	0	0.0

**Table 2:** Multiple linear regression results with the EQ-5D-5L at 6 months as the dependent variable.

Dependent Variable: EQ5D (6 Months)	β +	SE ++	*p*
Gender (women vs. men)	0.001	0.026	0.975
Age	−0.004	0.001	**0.001**
BMI			
Normal vs. obese	−0.036	0.036	0.328
Overweight vs. obese	0.042	0.033	0.211
Educational level			
Primary/secondary/high school vs. postgraduate degree	−0.020	0.041	0.628
Two years of college/university vs. postgraduate degree	−0.046	0.038	0.227
Married (yes vs. no)	0.016	0.025	0.523
Employed (yes vs. no)	−0.083	0.045	0.059
SSS-8 (pre)	−0.002	0.003	0.441
HADS depression (pre)	−0.008	0.004	0.077
HADS anxiety (pre)	−0.005	0.004	0.266
PMDQ score (pre)	−0.005	0.003	0.140
PNRS (pre)	−0.027	0.007	**<0.001**

Notes: Logarithmic transformations of the dependent variable were used for this analysis. +: regression coefficient; ++: standard error; SSS-8: Somatic Symptom Scale-8; HADS: Hospital Anxiety and Depression Scale; RMDQ: Rolland–Morris Disability Questionnaire; PNRS: pain numerical rating scale; EQ-5D-5L: EuroQol-5D-5-level. Bold: significant difference.

**Table 3:** Multiple linear regression results with the HADS depression and anxiety scales at 6 months as dependent variables.

Dependent Variable	Independent Variables	β +	SE ++	*p*
HADS Depression (6 months)	Gender (women vs. men)	0.039	0.052	0.450
	Age	0.001	0.003	0.752
	BMI			
	Normal vs. obese	0.018	0.070	0.793
	Overweight vs. obese	−0.071	0.064	0.272
	Educational level			
	Primary/secondary/high school vs. postgraduate degree	0.005	0.079	0.949
	Two years of college/university vs. postgraduate degree	0.003	0.073	0.973
	Married (yes vs. no)	0.008	0.049	0.869
	Employed (yes vs. no)	−0.047	0.081	0.562
	SSS-8 (pre)	0.018	0.006	**0.003**
	RMDQ (pre)	0.004	0.006	0.558
	PNRS (pre)	−0.008	0.013	0.540
HADS Anxiety (6 months)	Gender (women vs. men)	0.041	0.057	0.477
	Age	−0.002	0.003	0.562
	BMI			
	Normal vs. obese	0.068	0.077	0.375
	Overweight vs. obese	−0.022	0.070	0.757
	Educational level			
	Primary/secondary/high school vs. postgraduate degree	−0.090	0.087	0.305
	Two years of college/university vs. postgraduate degree	−0.078	0.081	0.337
	Married (yes vs. no)	0.017	0.054	0.748
	Employed (yes vs. no)	−0.060	0.089	0.500
	SSS-8 (pre)	0.017	0.006	**0.008**
	RMDQ (pre)	0.011	0.007	0.094
	PNRS (pre)	0.003	0.014	0.843

Note. Logarithmic transformations of the dependent variables were used for these analyses. +: regression coefficient; ++: standard error; SSS-8: Somatic Symptom Scale-8; HADS: Hospital Anxiety and Depression Scale; RMDQ: Rolland–Morris Disability Questionnaire; PNRS: pain numerical rating scale. Bold: significant difference.

**Table 4:** Multiple linear regression results with RMDQ Score, PNRS and SSS-8 scales at 6 months as dependent variables.

Dependent Variable	Independent Variables	β +	SE ++	*p*
RMDQ Score (6 months)	Gender (women vs. men)	−0.029	0.064	0.655
	Age	0.009	0.003	**0.006**
	BMI			
	Normal vs. obese	−0.060	0.087	0.492
	Overweight vs. obese	−0.140	0.079	0.078
	Educational level			
	Primary/secondary/high school vs. postgraduate degree	0.022	0.098	0.825
	Two years of college/university vs. postgraduate degree	0.101	0.090	0.264
	Married (yes vs. no)	−0.020	0.061	0.742
	Employed (yes vs. no)	0.170	0.100	0.091
	SSS-8 (pre)	0.016	0.007	**0.025**
	PNRS (pre)	0.036	0.016	**0.024**
PNRS (6 months)	Gender (women vs. men)	−0.002	0.047	0.963
	Age	0.001	0.002	0.723
	BMI			
	Normal vs. obese	0.001	0.062	0.993
	Overweight vs. obese	0.007	0.057	0.905
	Educational level			
	Primary/secondary/high school vs. postgraduate degree	0.013	0.072	0.853
	Two years of college/university vs. postgraduate degree	0.027	0.066	0.683
	Married (yes vs. no)	0.000	0.045	0.993
	Employed (yes vs. no)	−0.082	0.073	0.262
	SSS-8 (pre)	0.019	0.005	**<0.001**
SSS-8 (6 months)	Gender (women vs. men)	0.094	0.049	0.057
	Age	0.001	0.002	0.703
	BMI			
	Normal vs. obese	−0.113	0.064	0.082
	Overweight vs. obese	−0.139	0.060	**0.023**
	Educational level			
	Primary/secondary/high school vs. postgraduate degree	−0.016	0.077	0.834
	Two years of college/university vs. postgraduate degree	−0.006	0.071	0.932
	Married (yes vs. no)	−0.014	0.048	0.765
	Employed (yes vs. no)	−0.024	0.078	0.757

Note. Logarithmic transformations of the dependent variables were used for these analyses. +: regression coefficient; ++: standard error; SSS-8: Somatic Symptom Scale-8; RMDQ: Rolland–Morris Disability Questionnaire; PNRS: pain numerical rating scale. Bold: significant difference.

**Table 5:** Changes in the study’s scale values throughout the follow-up period.

	Pre ^1^		Post ^2^		6 Months ^3^		P Friedman Test	Effect Size W
	Mean (SD)	Median (IQR)	Mean (SD)	Median (IQR)	Mean (SD)	Median (IQR)		
SSS-8 score	9.4 (4.67) ^2,3^	9 (6–12)	6.92 (4.03) ^1,3^	6 (4–10)	8.2 (4.9) ^1,2^	8 (4–11)	<0.001	0.15
HADS depression score	5.87 (3.3)	6 (3–8)	5.44 (3.27)	5 (3–7)	5.63 (3.61)	5 (3–8)	0.059	0.02
HADS anxiety score	5.41 (3.52) ^2^	6 (2–7)	4.76 (3.54) ^1^	4 (2–7)	5.22 (3.6)	5 (3–7)	0.002	0.05
RMDQ	7.67 (4.59) ^2,3^	7 (4–10)	5.52 (4.39) ^1^	5 (2–8)	5.95 (5.24) ^1^	4 (1–9)	<0.001	0.15
EQ-5D-5L value	0.68 (0.16) ^2,3^	0.71 (0.6–0.78)	0.75 (0.15) ^1^	0.76 (0.68–0.86)	0.72 (0.18) ^1^	0.76 (0.68–0.83)	<0.001	0.20
PNRS (0–10 scale)	4.73 (2.11) ^2,3^	5 (3–6)	2.93 (1.97) ^1,3^	3 (2–5)	3.58 (2.2) ^1,2^	3 (2–5)	<0.001	0.30

^1,2,3^ indicate significant differences after the Bonferroni correction; SSS-8: Somatic Symptom Scale-8; HADS: Hospital Anxiety and Depression Scale; RMDQ: Rolland–Morris Disability Questionnaire; PNRS: pain numerical rating scale; EQ-5D-5L: EuroQol-5D-5-level.

## Data Availability

The data are available upon reasonable request.

## Abbreviations

The following abbreviations are used in this manuscript:

SSD	Somatic symptom disorders
CLBP	Chronic low back pain
PNRS	Pain numerical rating scale
RMDQ	Roland–Morris Disability Questionnaire
EQ-5D-5L	EuroQol-5-dimension-5-level
HADS	Hospital Anxiety and Depression Scale
SSS-8	Somatic Symptom Scale-8
HRQoL	Health-related quality of life
TENS	Transcutaneous electrical nerve stimulation
US	Ultrasound
TYPET	Greek acronym for the Mutual Fund of National Bank of Greece Personnel
BMI	Body mass index
